# A Path-Based Partial Information Decomposition

**DOI:** 10.3390/e22090952

**Published:** 2020-08-29

**Authors:** David Sigtermans

**Affiliations:** ASML, De Run 6501, 5504 DR Veldhoven, The Netherlands; david.sigtermans@asml.com

**Keywords:** information theory, causal inference, mutual information, transfer entropy, tensors, paths, partial information decomposition, data processing inequality, diagnostics

## Abstract

Based on the conceptual basis of information theory, we propose a novel mutual information measure—‘path-based mutual information’. This information measure results from the representation of a set of random variables as a probabilistic graphical model. The edges in this graph are modeled as discrete memoryless communication channels, that is, the underlying data is ergodic, stationary, and the Markov condition is assumed to be applicable. The associated multilinear stochastic maps, tensors, transform source probability mass functions into destination probability mass functions. This allows for an exact expression of the resulting tensor of a cascade of discrete memoryless communication channels in terms of the tensors of the constituting communication channels in the paths. The resulting path-based information measure gives rise to intuitive, non-negative, and additive path-based information components—redundant, unique, and synergistic information—as proposed by Williams and Beer. The path-based redundancy satisfies the axioms postulated by Williams and Beer, the identity axiom postulated by Harder, and the left monotonicity axiom postulated Bertschinger. The ordering relations between redundancies of different joint collections of sources, as captured in the redundancy lattices of Williams and Beer, follow from the data processing inequality. Although negative information components can arise, we speculate that these either result from unobserved variables, or from adding additional sources that are statistically independent from all other sources to a system containing only non-negative information components. This path-based approach illustrates that information theory provides the concepts and measures for a partial information decomposition.

## 1. Introduction

To understand and to *diagnose* changes in the behavior of a specific component of a complex system, we need to explain the behavior of this specific component in terms of the behavior of the other components in that complex system, the sources, and combination of these components, that is, sets of sources. This interconnectedness of behaviors can be described with information theory [1]. Information theory also gives rise to a succinct description of the interrelations in a multivariate complex system as a probabilistic graphical model—an edge represents an unidirectional communication channel between a source and a destination, and the “nodes”, or vertices, are the sources and/or destinations.

An issue with information theory is that it does not provide a measure to capture multivariate mutual information. Because of this, a multivariate extension of mutual information, “interaction information”, was introduced [2]. This signed measure is widely used, despite difficulties with interpreting the results when applied to systems comprising over three variables. Recent advances have been made in both our understanding of interaction information [3], and with respect to its applicability to systems comprising over three variables [4]. However, these advances do not resolve a fundamental problem with the measures provided by, and/or based on “classical” information theory. In Reference [5] it is illustrated that interaction information, and many other information measures, are incapable to differentiate between two multivariate systems, both comprising three random variables with identical joint probabilities, but with different underlying dependency structures. Because of this, we focus in this paper on the “partial information decomposition” (PID), proposed by Williams and Beer [6] as an alternative to interaction information.

The total information shared between the target and the rest of the network is broken down into three additive, non-negative information contributions—*unique*, *redundant*, and *synergistic* information. Unique information is information contributed by one and only one specific source, that is, information that is only shared between one specific source and the target. Redundant information is information that is shared between multiple sources and the target, while synergistic information is neither unique, nor redundant and shared between the target and a set of sources. A foundational notion in the PID is that redundancy between a source and any superset, that is, any set of sources containing that source, is equal to the information of that source. This results in an ordering relation between the collections, guaranteeing non-negativity. The ordering relation is captured in the “redundancy lattice” [6]. While the lattice is widely accepted [7], the proposed redundancy term, $I_{min}$ is not. Again information theory seems to be incomplete, for a system comprising three variable, there are three equations that connect four information terms, one redundant information term, two unique and terms and one synergistic information contribution. The freedom in defining redundant information, unique information of synergistic information, led to several definitions, each with their own idiosyncrasies [8]. In the search for a potentially ultimate answer, new axioms are proposed by for example Harder [9] and Bertschinger et al. [10]. It is generally accepted that the standard measures provided by information theory, for example, mutual information (MI), are insufficient. This is demonstrated by, for example, Ince [11], and Finn and Lizier [12,13]: using aggregate measures to capture internal dynamics leads to unsatisfactory results.

In this paper, we contribute to the current discourse by showing that “classical information theory” does deliver the concepts and framework to quantify the distinct information components used in the PID. For this we define a new type of mutual information, path-based mutual information. Using this path-based information, a path-based redundancy is defined. This path-based redundancy satisfies the three axioms postulated by Williams and Beer by definition. It also satisfies the identity property postulated by Harder [9], and the left monotonicity axiom postulated by Bertschinger et al. [10]. The ordering relations between the redundancies of different joint collections of sources, as captured in the redundancy lattices, result from the application of the data processing inequality [14]. The path-based PID, and the related tensor representation are both capable to differentiate between the two multivariate systems from Reference [5].

The structure of this article is as follows—in Section 2 tensors, a generalization of transition probability matrices representing discrete memoryless communication channels, and path-based mutual information are introduced. These concepts are foundational to our proposed definition of path-based redundant and unique information. In Section 2.3 it is shown that the proposed path-based redundancy meets the three axioms postulated by Williams and Beer [6], and the earlier mentioned additional axioms postulated by Harder [9] and Bertschinger et al. [10]. As a consequence of the proposed definitions and the chosen notation for paths, the reader will immediately recognize that the lattice elements of the redundancy lattice can be interpreted as paths. This allows for the conclusion that the lattice reflects the data processing inequality. We furthermore provide a potential explanation for demonstrated negative synergistic contributions. In Section 3, the proposed PID is applied to some standard systems.

## 2. Materials and Methods

We make similar assumptions as Shannon [1]: (i) the data are realizations of random variables representing stationary ergodic processes, (ii) the Markov Condition is applicable, that is, a process is independent of its non-effects given its direct causes [15], and (iii) the data are drawn from a finite alphabet, allowing for the use of indices when a fixed order of the alphabet elements is assumed. A random variable is denoted by an uppercase letter, for example, *X*, while its realizations are denoted by a lowercase letter, for example, *x*. With respect to the systems under consideration, we assume that if there is a path between two vertices, there is *at least* one other path between these vertices, unless stated otherwise.

The main idea behind the our approach is that unique information must come from a source that is capable to (also) transmit its data directly to the destination, and that redundant information is therefore transferred via paths with lengths greater than one. In information theory, association between data is *modeled* as the result of transmission of data over a (conceptual) communication channel. Due to the Markovian nature of the system, the communication channels are discrete and memoryless [14]. A communication channel transforms the probability mass function (pmf) of the source data, represented as row stochastic vector, in the pmf of the destination data via a linear mapping. A communication channel is fully characterized by a transition probability tensor [14]. This means that linear algebra can be used to give an expression for the transition probability tensor resulting from a cascade, or path, in terms of tensors constituting the cascade. This leads to a proposal for a path-based redundancy, resulting in a path-based partial information decomposition.

### 2.1. Tensors

The linear mapping becomes apparent when the probabilities are represented as tensors [16]. In this article we use contra-variant and covariant index notation. This notation allows for the use of the variable names as indices in the case of tensors. The covariant or lower index indicates the conditioning variable, while the contra-variant or upper index indicates the variable that is conditioned. Values for the random variable *X* are selected from alphabet X, and values for random variable *Y* are selected from alphabet Y. Index *x* indicates the $x^{th}$ alphabet symbol of X, and *y* indicates the $y^{th}$ alphabet symbol of Y respectively. For example, p(y|x) equals the probability Y=y given X=x. This is equal to the tensor element $p_{x}^{y}$, which equals the probability of $y^{th}$ alphabet element of Y, given the $x^{th}$ alphabet element of X. Because the tensors are assumed to be constant and representative for the communication channels, uppercase letters are used, for example, the tensor A represents the association X→Y. In a similar fashion, probability p(x) can be represented as the row vector $p^{x}$, and p(y) as the row vector $p^{y}$. For a memoryless channel, the relation between the source pmf and the pmf of the destination can now be written as

(1) $$p^{y}=\sum_{x}p^{x}A_{x}^{y},$$

with $A_{x}^{y}$ a component of the tensor A. A communication channel is the conceptual implementation of the Law of Total Probability [17]. This implies that the source pmf can be reconstructed from the destination pmf,

$$p^{x}=\sum_{y}p^{y}A_{y}^{‡x},$$

where the “‡” symbol indicates that the source pmf is reconstructed. Any association, that is, mutual information [14], between variables, can be thought of as resulting from transmission of data via a communication channel. The mutual information between the source variable *X*, and the destination variable *Y*, is usually expressed as

(2) $$I\left(X;Y\right)\;=\;\sum_{x\in X,y\in Y}p\left(x,y\right)\log_{2}\left[\frac{p(y|x)}{p(y)}\right].$$

Using tensor notation, Equation (2) can also be written as

(3) $$I\left(X;Y\right)=\sum_{x,y}p^{xy}\log_{2}\left[\frac{A_{x}^{y}}{p^{y}}\right],$$

with $p^{xy}\;=\;p\left(x,y\right)$. Because the right-hand side contains tensors, *x* and *y* should be interpreted as indices.

#### Paths and Tensors of a Cascade

As mentioned in the introduction, a system comprising more than one random variable is represented by a graph. The vertices represent random variables, and the (undirected) edges represent associations. The probability distributions are only defined for these edges. For any path in the graph, a sequence of vertices where each vertex in the sequence is adjacent to the previous vertex [18], the resulting probability distribution can be calculated. The transition probability tensor between the first vertex and the last vertex of a path is the product of all the tensors of the direct associations constituting the path. For example, the transition probability tensor C describing the transformation from the pmf of *X* to the pmf of *Z*, resulting from the chain X→Y→Z, equals

(4) $$C_{x}^{z}=\sum_{y}A_{x}^{y}B_{y}^{z}\;,$$

where tensor B represents the transformation Y→Z. In Appendix A it is proven that this relation is also valid in case there are more variables in the system, and when *X* and *Z* are not independent given *Y*. To distinguish between different paths, a specific notation for the path $P_{i}$ between a source and a destination is chosen,

$$P_{i}=\left\{source\right\}\;\left\{mediator_{1}\right\}\;\cdots \;\left\{mediatior_{n}\right\}\;\left\{destination\right\}.$$

The index *i* is needed in case there are multiple paths starting at {source} and ending at {destination} that contain the same, but permuted mediators. In the remainder of the paper, the mediators are also called sources. To simplify the notation for the resulting tensor for a path, T is added as a prefix. For example, T{Y}{Z} is the tensor resulting from the path {Y}{Z}. In the rest of this article we, sometimes, indicate the tensors of specific paths by their “name”: A:=T{X}{Y}, B:=T{Y}{Z}, $A^{‡}\;:=\;T\left\{Y\right\}\left\{X\right\}$, $B^{‡}\;:=\;T\left\{Z\right\}\left\{Y\right\}$, C:=T{X}{Z}, and $C^{‡}\;:=\;T\left\{Z\right\}\left\{X\right\}$. As per Equation (4), it follows that

(5) T{X}{Y}{Z}=T{X}{Y}·T{Y}{Z}.

### 2.2. Path-Based Mutual Information

In the previous section, paths and their related tensors were introduced. In this section path-based mutual information is introduced. Path-based mutual information is a measure of association between the source and the destination of a path. Applying Equation (5) to the graph X→Y→Z, the mutual information for the path {X}{Y}{Z} equals

(6) $$I_{p_{1}}\left(X;Z\right)=\sum_{x,z}p^{xz}\log_{2}\left[\frac{\sum_{y}A_{x}^{y}B_{y}^{z}}{p^{z}}\right],$$

where the index $p_{1}$ indicates that the probability distributions are associated with path {X}{Y}{Z}. Based on this example we defined the path-based mutual information for any path length.

**Definition** **1** (Path-Based Mutual Information). *Let $\left\{X_{1}\right\}\left\{X_{2}\right\}\cdots \left\{X_{i}\right\}\left\{X_{i+1}\right\}\cdots \left\{X_{\ell }\right\}$ be a path of length ℓ−1, and let $M_{i}^{i+1}$ represent the tensor, for the path $\left\{X_{i}\right\}\left\{X_{i+1}\right\}$. The tensor for the whole path equals*

$$T=\prod_{i=1}^{\ell -1}M_{i}^{i+1}.$$

*The path-based mutual information is defined as*

$$I\left\{X_{1}\right\}\left\{X_{2}\right\}\cdots \left\{X_{\ell }\right\}=\sum_{x_{1},x_{\ell }}p^{x_{1}x_{\ell }}\log_{2}\left[\frac{T_{x_{1}}^{x_{\ell }}}{p^{x_{\ell }}}\right].$$

*The left-hand side can also be written as $I\left\{X_{1}\right\}\left\{X_{2}\right\}\cdots \left\{X_{\ell }\right\}\;=\;I_{p_{1}}\left(X_{1};X_{\ell }\right)$, where $p_{1}$ indicates that the probability distributions are associated with the path $\left\{X_{1}\right\}\left\{X_{2}\right\}\cdots \left\{X_{\ell }\right\}$.*

To develop some intuition for the relationship between traditional MI and path-based mutual information, we study a system comprising four variables, represented by the graph X→Y→Z and X→W→Z. Because there are two paths between *X* and *Z*, in general $I_{p_{1}}\left(X;Z\right)\;\neq \;I\left(X;Z\right)$. This becomes clear when using the standard notation for mutual information in terms of probabilities instead of tensors. Equation (6) can be written as

(7) $$I_{p_{1}}\left(X;Z\right)=\sum_{x\in X,z\in Z}p\left(x,z\right)\log_{2}\left[\frac{\sum_{y\in Y}p\left(y|x\right)p\left(z|y\right)}{p(z)}\right].$$

When *Z* and *X* are independent given *Y* it follows that $p\left(z|x\right)\;=\;\sum_{y}p\left(y|x\right)p\left(z|y\right)$. Equation (7) reduces to the expression for I(X;Z),

(8) $$I_{p_{1}}\left(X;Z\right)\;=\;\sum_{x\in X,z\in Z}p\left(x,z\right)\log_{2}\left[\frac{p(z|x)}{p(z)}\right].$$

When *Z* and *X* are dependent given *Y*, that is, in the case of more than one path between *X* and *Z*, $I\left(X;Z\right)\;\neq \;I_{p_{1}}\left(X;Z\right)$. The equality between traditional and path-based mutual information in case there is only one path between the source and destination, enables us to express mutual information in terms of path-based mutual information. For example, the MI between *X* and *Y*, equals $I\left(X;Y\right)\;=\;I_{p_{1}}\left(X;Y\right)$, with $p_{1}$ the probability distributions associated with the path {X}{Y}. Multivariate MI, for example, I(X,Y;Z), can be expressed as $I\left(X,Y;Z\right)\;=\;I_{p_{1}}\left(X,Y;Z\right)$, with $p_{1}$ the probability distributions associated with the path {XY}{Z}, and {XY} representing the joint sources. Additionally, all of the MI equalities and inequalities can be expressed in terms of path-based mutual information. For example, the chain rule for mutual information [14],

$$I\left(X_{1},X_{2},\cdots ,X_{n};Z\right)=\sum_{i=1}^{n}I\left(X_{i};Z|X_{1},X_{2},\cdots ,X_{i-1}\right),$$

can also be written as

$$I\left\{X_{1}X_{2}\cdots X_{n}\right\}\left\{Z\right\}=\sum_{i=1}^{n}I\left\{X_{i}|X_{1},X_{2},\cdots ,X_{i-1}\right\}\left\{Z|X_{1},X_{2},\cdots ,X_{i-1}\right\}.$$

Applied to a system comprising three nodes, the chain rule leads this leads to the following well-known inequality

(9) I{XY}{Z}≥max[I{X}{Z},I{Y}{Z}].

Up till now, only trivial, notational aspects were discussed. However, path-based MI also has some non-trivial implications.

#### 2.2.1. Some Non-Trivial Aspects of Path-Based Mutual Information

In the previous section it was mentioned that when there are two or more different paths between *X* and *Z*, $I\left(X;Z\right)\;\neq I_{p_{i}}\;\left(X;Z\right)$ where the index $p_{i}$ indicates that the probability distributions are associated with the $i^{th}$ path. Traditional mutual information is bounded by the minimum over the path-based mutual informations. The relationship between path-based mutual information and traditional MI for a system with *P* different paths between, say, *X* and *Z*, equals

(10) $$I\left(X,Z\right)\geq \min\left[\underset{i\in \{1,\cdots ,P\}}{⋃}\left\{I_{p_{i}}\left(X;Z\right)\right\}\right],$$

where the set of path-based mutual informations for all paths between *X* and *Z*, $\left\{I_{p_{1}}\left(X;Z\right),\cdots ,I_{p_{P}}\left(X;Z\right)\right\}$, is written as ${⋃}_{i\in \{1,\cdots ,P\}}\left\{I_{p_{i}}\left(X;Z\right)\right\}$, with ⋃ the union operator. To arrive at the lower bound, we use the example of a graph comprising four vertices and the paths X→Y→Z and X→W→Z. First lets assume that Y=W. In this trivial case, $I\left(X;Z\right)\;=\;\min\left[I_{p_{1}}\;\left(X;Z\right),I_{p_{2}}\;\left(X;Z\right)\right]$. The indices $p_{1}$ and $p_{2}$ indicate that the probability distributions are associated with path {X}{Y}{Z} and path {X}{W}{Z} respectively. Changing one of the mediators, for example, *Y*, so that the path-based mutual information increases for that path, can’t result in a decrease of the mutual information between *X* and *Z*, that is, $I\left(X;Z\right)\;\geq \;\min\left[I_{p_{1}}\;\left(X;Z\right),I_{p_{2}}\;\left(X;Z\right)\right]$.

Another relation between path-based MI and traditional mutual information is related to the symmetry of MI, I(X;Y)=I(Y;X). In Appendix B, it is proven that the mutual information of a path is *traverse invariant*. The symmetry of MI is a special case of this invariance.

**Lemma** **1.** *The mutual information of a path equals the mutual information of the path traversed in the opposite direction. The MI of a path is traverse invariant*

(11) $$I_{p_{1}}\left(X;Z\right)=I_{p_{1}}\left(Z;X\right),$$

*where $p_{1}$ indicates that the probability distributions are associated with paths {X}{Y}{Z} and {Z}{Y}{X}.*

Equation (4) is valid in any Markovian system comprising three or more variables, therefor, Equation (6) is also valid in any Markovian system comprising three or more variables. This gives rise to the data processing inequality (DPI) [14] for paths.

**Theorem** **1** (Data Processing Inequality for Paths). *For a system comprising three variables*

(12) $$I_{p_{1}}\left(X;Z\right)\;\leq \;\min\left[I\left(X;Y\right),I\left(Y;Z\right)\right],$$

*with $p_{1}$ the probability distributions associated with path {X}{Y}{Z}.*

The proof follows directly from Equation (4). The inequality resulting from the chain rule for mutual information, Equation (9), can also be interpreted as resulting from the DPI. In Reference [19], it is proven that for a system comprising three variables the following relations hold T{X}{Z}=T{X}{XY}{Z}, and T{Y}{Z}=T{Y}{XY}{Z}.

#### 2.2.2. Indirect Associations and No Associations

There are two underlying reasons to delete an edge in a graph: (i) there is no association at all, or (ii) the association it represents is the result of an indirect path. These two reasons can be distinguished using tensors. First, if there is no association between two vertices at all, the tensor represents a communication channel that cannot transmit any information. In this case the transition probability tensor has identical rows, for example, $\forall \;x^{\prime }\neq x:\;A_{x^{\prime }}^{y}=A_{x}^{y}$. Second, the resulting tensor of a cascade can be determined exactly from the constituting tensors. This enables the differentiation between direct and indirect associations using bivariate measurements [19].

**Proposition** **1.** *If the association between two vertices results from an indirect association, or if the association does not exist, the direct path does not exist. For example, the path {X}{Z} does not exist when the graph X→Y→Z is the ground truth.*

### 2.3. Partial Information Decomposition

The partial information decomposition framework of Williams and Beer [6] allows for a decomposition of the total information in non-negative unique, redundant, and synergistic information components. The unique information U(Y;Z) represents information in *Z* only provided by *Y* and not by *X*. The redundant information R(X,Y;Z) represents the information in *Z* provided by both *X* and *Y*. The synergistic information S(X,Y;Z) represents information in *Z* that results via interaction between *X* and *Y*. The relations between these information components for a system comprising three variables are given by the following set of equations,

(13) $$\begin{array}{cc} I(X,Y;Z) & =U(Y;Z)+U(X;Z)+R(X,Y;Z)+S(X,Y;Z), \end{array}$$

(14) $$\begin{array}{cc} I(Y;Z) & =U(Y;Z)+R(X,Y;Z), \end{array}$$

(15) $$\begin{array}{cc} I(X;Z) & =U(X;Z)+R(X,Y;Z). \end{array}$$

Apart from the additivity, Williams and Beer propose three redundancy related axioms: (i) Symmetry: redundancy does not change when sources are permuted, for example, R(X,Y;Z)=R(Y,X;Z). (ii) self-redundancy: for a single source, the redundancy equals the mutual information between the source and the target, for example, R(X,X;Z)=I(X;Z). From now on we set R(X,X;Z)=R(X;Z) (iii) monotonicity: the redundancy does not increase when a new source is added, for example, R(X;Z)≥R(X,Y;Z).

The definition of redundant information as the information that is also shared between sources, implies that redundant information cannot exceed the mutual information between the related sources. In the case of two sources, R(X,Y;Z)≤I(X;Y). Assuming that the unique information is non-negative, it follows from Equations (14) and (15) that

(16) $$R\left(X,Y;Z\right)\leq \min\left[I(X;Y),I(X;Z),I(Y;Z)\right].$$

In other words, Equation (16) implies non-negativity of unique information, and non-negativity of unique information implies Equation (16).

#### Path-Based Redundancy, Indirect Paths and the Data Processing Inequality

If the association between two vertices is indirect, no direct path exists. Source data is transmitted to the next source, during which data is possibly modified and/or stored, after which it is transmitted again towards the next source or destination, that is, the target. This consideration leads to the following proposition:

**Proposition** **2.** *Path-based unique information can only result from data transmission via a direct path. Path-based redundant information results from data transmission via an indirect path.*

A direct consequence of this proposition is that in case the chain X→Y→Z is the ground truth, no unique information is shared between *X* and *Z*, or stated otherwise, all information shared between *X* and *Z* is redundant. Another immediate consequence of this proposition is that in case of the exclusive or example from Reference [20], there is neither unique, nor redundant information shared between the sources and the target—all information shared is synergistic.

To express path-based redundancy in terms of path-based mutual information, lets consider a fully connected three-node system. In this case there are two source vertices transmitting data to the target vertex, and, per source vertex, there is one indirect path between that source vertex and the target vertex. Assume that *Z* is the target. According to Equation (14), unique information from *Y* and shared information between *X* and *Y* is transmitted towards *Z* via the path {X}{Y}{Z}. The DPI applied to the path {X}{Y}{Z} results in the inequality

$$I_{p_{1}}\left(X;Z\right)\;\leq \;\min\left[I\left(X;Y\right),I\left(Y;Z\right)\right],$$

with $p_{1}$ the probability distributions associated with path {X}{Y}{Z}. Similarly, the target *Z* receives unique information from *X*, and shared information between *X* and *Y* via the path {Y}{X}{Z}. The DPI applied to this path results in

$$I_{p_{2}}\left(Y;Z\right)\;\leq \;\min\left[I\left(Y;X\right),I\left(X;Z\right)\right],$$

with $p_{2}$ the probability distributions associated with path {Y}{X}{Z}. Combining both DPI related inequalities results in

(17) $$\min\left[I_{p_{1}}\left(X;Z\right),I_{p_{2}}\left(Y;Z\right)\right]\leq \min\left[I(X;Y),I(X;Z),I(Y;Z)\right].$$

Comparison with Equation (16), leads to our proposition for *path-based redundancy*.

**Definition** **2** (Path-Based Redundancy). *Path-based redundant information shared between a specific set of sources with respect to a target is defined as the minimum path-based information over all indirect paths that: (1) contain all the sources, (2) start with a source and ends at the target, and (3) do not contain non-existing paths. For a system comprising ℓ sources, path-based redundancy is defined as*

(18) $$R\left(X_{1},\cdots ,X_{\ell };Z\right)=\min\left[\underset{k\in \{1,\cdots ,\ell \}}{⋃}\left\{I_{p_{ik}}\left(X_{k};Z\right)|i\in \left\{1,\cdots ,N_{k}\right\}\right\}\right],$$

*with $p_{ik}$ the probability distribution over the $i^{th}$ path starting at $X_{k}$ and ending at Z.*

The reader can verify that for a system comprising three variables

(19) $$R\left(X,Y;Z\right)=\min\left[I_{p_{1}}\left(X;Z\right),I_{p_{2}}\left(Y;Z\right)\right],$$

with $p_{1}$ and $p_{2}$ the probability distributions associated with paths {X}{Y}{Z} and {Y}{X}{Z} respectively.

Please note that Equation (17) combined with (19), leads to an equation that is identical to the redundancy definition used in Reference [21].

### 2.4. Characteristics of Path-Based Redundancy

In this section some of the characteristics of the proposed path-based redundancy will be presented. We start with the proofs of the three axioms postulated by Williams and Beer [6].

#### 2.4.1. Symmetry, Self-Redundancy, and Monotonicity

**Proof** **of** **Symmetry.** Symmetry follows directly from Equation (18). Permuting the sources on the left-hand side of Equation (18), does not affect the right-hand side. The right-hand side contains the path-based mutual informations of all possible paths containing all the sources, that is, the order of the sources in the left-hand side is irrelevant. □

**Proof** **of** **Self-Redundancy.** Consider a system consisting of three variables, the sources *X* and *Y*, and the target *Z*. Let now assume that one source is a copy of the other source, that is, Y=X. This means that the tensors describing the mapping between the sources and target, T{Y}{Z} and T{X}{Z}, are equal: T{Y}{Z}=T{X}{Z}. By definition R(X,X;Z)=I{X}{X}{Z}. The tensor for the path {X}{X}, T{X}{X}, equals the Kronecker delta $\delta_{x}^{x}$: $\delta_{x}^{x}\;=\;0$ unless x=x in which case $\delta_{x}^{x}\;=\;1$. Therefore, using Equation (6), the tensor of the path {X}{X}{Z} equals the tensor of the path {X}{Z}, that is, I{X}{X}{Z}=I(X;Z), in other words, for a single source, the path-based redundancy equals the mutual information between the source and the target. □

**Proof** **of** **Monotonicity.** Because of the definition of redundancy, adding more sources never decreases the number of edges in the cascade. As per data processing inequality, adding sources can therefore never increase the redundancy: $R\left(X_{1},\cdots ,X_{\ell };Z\right)\;\geq \;R\left(X_{1},\cdots ,X_{\ell },X_{\ell +1};Z\right)$. □

#### 2.4.2. Generalized Self-Redundancy and the Path-Based Redundancy Inequality

The proof of self-redundancy uses the fact that copying a variable is equivalent to a noiseless data transmission. The tensor for this transmission is the Kronecker delta. The Kronecker delta is a special case of a permutation tensor, the rows and columns contain exactly one non-zero entry. The self-redundancy axiom is a special case of a *generalized self-redundancy*. It uses a specific definition of equivalence.

**Definition** **3.** *Two random variables are equivalent if one random variable is the result of a noiseless transmission of the other variable.*

**Theorem** **2** (Generalized Self-Redundancy). *If Y results from a noiseless data transmission of X, that is, Y and X are equivalent, then R(X,Y;Z)=I(X;Z).*

This theorem is proven in Appendix D. From this proof it immediately follows that

**Corollary** **1.** *If Y results from a noiseless data transmission of X, that is, Y and X are equivalent, then R(Z,Y;X)=R(Z,X;Y). If X and Y are not equivalent, R(Z,Y;X)≠R(Z,X;Y).*

In Appendix E, it is proven that within a system comprising three variables there is a specific relation between the redundancies within this system.

**Theorem** **3** (Path-Based Redundancy Inequality).

(20) $$R\left(X,Y;Z\right)\geq \min\left[R(Z,Y;X),R(Z,X;Y)\right].$$

#### 2.4.3. The Redundancy Lattice, Paths and the DPI

**Figure 1:**
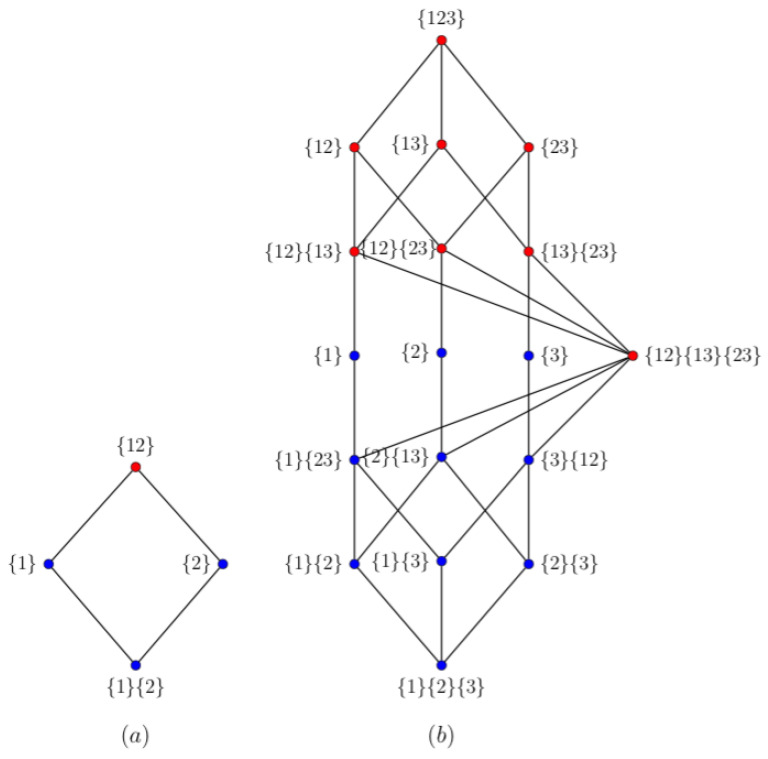
The redundancy lattice [6] for: (**a**) two sources, and (**b**) three sources. The labels next to the vertices indicate the collection of (joint) sources. In case two lattice vertices are connected, the redundancy related to the highest lattice vertex (in position) is greater than or equal to the redundancy of the lower lattice vertex. The ordering relation for the vertices of the same color follows directly from the Data Processing Inequality.

In Figure 1 the two redundancy lattices from Reference [6] are reproduced. Each vertex is also associated to a *partial information atom*. The related redundancies can be considered as resulting from a cumulative information function over the partial information atoms. These lattices therefore reflect the ordering relation in the redundancy. To determine the value of each partial information atom using the redundancies, the recursive relationship (Möbius inverse) over the redundancy values of the lattice should be used [6,11].

When we interpret the nodes of the lattice as path redundancies, the ordering relation is invoked by the DPI. First we notice that the target vertex is omitted in the lattice labels. If we would add the target vertex, say {t}, the labels represent all the paths resulting from permutation of the sources. For example, the vertex with the label {1}{2} in Figure 1a, represents the redundancy related to the paths {1}{2}{t} and {2}{1}{t}. The vertex with the label {1} represents the redundancy related to the path {1}{t}. A direct consequence of Self-Redundancy is that Equation (16) can be rewritten as

(21) $$R\left(X,Y;Z\right)\leq \min\left[R(X;Y),R(X;Z),R(Y;Z)\right],$$

Because of the definition of path-based redundancy, Equation (21) is a consequence of the data processing inequality. Therefor, the ordering relation between the two levels of blue vertices in Figure 1a is also a consequence of the DPI. The ordering relation between the top red vertex labeled {12}, and the two blue vertices labeled {1} and {2} follow from Equation (9), R(12;t)≥max[R(1;t),R(2;t)]. To summarize, for a system comprising three variables, the path-based redundancy is subjected to ordering relations that match the redundancy lattice derived in Reference [6]: R(1,2;t)≤R(1;t), R(1,2;t)≤R(2;t), R(1;t)≤R(12;t), and R(2;t)≤R(12;t). For a system comprising four variables, see Figure 1b, the ordering relations also result from the DPI. For the identical colored vertices, the DPI is directly applicable. The DPI for a path implies that addition of a node at the beginning or end of a path does not increase the mutual information between the source and destination of that path as compared to the original path. Using this principle it, is proven in Appendix C that

(22) I{12}{13}{t}≥I{1}{t}.

The ordering relation between connected blue and red vertices, for example, {1} and {12}{13} in Figure 1b, is therefore also considered as resulting from the DPI.

#### 2.4.4. Identity and Left Monotonicity

Apart from these three axioms, Bertschinger et al. [10] and Harder et al. [9] proposed other properties. The proposed path-based redundancy measure satisfies the “identity property”. The intuition behind the identity property [9] is that if the target is a join of the inputs, the redundancy equals the mutual information of inputs, that is,

(23) R(X,Y;XY)=I(Y;X).

In Appendix F we prove that the proposed path-based redundancy property satisfies the identity property. The left monotonicity property captures the intuition that the redundancy does not *decrease* when a new destination is added [10]. In Appendix G it is proven that the proposed path-based redundancy satisfies the left monotonicity axiom

(24) $$R\left(X,Y;Z_{1}Z_{2}\right)\geq R\left(X,Y;Z_{1}\right).$$

#### 2.4.5. Path-Based Unique Information

Path-based unique information for a three-node system is defined by Equations (14) and (15). Using Definition 2 it follows immediately that the path-based unique information is non-negative. The resulting path-based unique information fully matches our intuition. This is demonstrated by the fully connected three-node system. Path-based redundant information in *Z* results from information transmitted via the paths {X}{Y}{Z} and {Y}{X}{Z}. The tensor elements of the path {Y}{X}{Z} and the path {X}{Y}{Z} are given by $\sum_{x}A_{y}^{‡x}C_{x}^{z}$ and $\sum_{y}A_{x}^{y}B_{y}^{z}$ respectively. Using Equation (14), the unique information equals

(25) $$U\left(Y;Z\right)=\max[\sum_{x,y,z}p^{xyz}\log_{2}[\frac{B_{y}^{z}}{\sum_{x^{\prime }}A_{y}^{‡x^{\prime }}C_{x^{\prime }}^{z}}],\;\sum_{x,y,z}p^{xyz}\log_{2}[\frac{B_{y}^{z}}{\sum_{y^{\prime }}A_{x}^{y^{\prime }}B_{y^{\prime }}^{z}}]].$$

The first sum is a measure for the divergence between the direct path {Y}{Z} and the indirect path {Y}{X}{Z}. If the *association* between *Y* and *Z* is indirect, this sum evaluates to zero, that is, the direct path between *Y* and *Z* does not exist. In this case the tensor B represents a non-existing communication channel: the second sum also equals zero.

The second sum is an indication of how much *Y* differs from *X*. If *Y* is an exact copy of *X* the tensors A and $A^{‡}$ both equal the Kronecker delta, this second sum evaluates to zero. In that case, B=C, that is, the first sum also vanishes. In other words, path-based information can only be unique if there is a direct path, as per Proposition 2, and if there is unique information to be conveyed, that is, both sources are not copies of each other.

## 3. Results

We start with investigating the behavior of the proposed partial information decomposition with respect the conceptual issue related to $I_{min}$, the original redundancy measure used in Reference [6]: $I_{min}$ does not distinguish between “same information” or “the same amount of information”, that is, it conflicts with the identity property [9]. We already know that the proposed path-based redundancy satisfies the identity relation, but this example familiarizes the reader with the method.

### 3.1. Two Bit Copy Problem

A conceptual problem with the redundancy measure used in Reference [6], $I_{min}$, is illustrated with the so called “two-bit copy problem”. For two independent and identically distributed binary variables *X* and *Y*, the target *Z* is a copy of these two variables: Z=(X,Y). It can be shown that $I_{min}\left(1,2;Z\right)\;=\;1$ bit [11]. The problem lies in the fact that there is no overlap between the information of both variables—the result does not match out intuition, $I_{min}$ seems to overestimate the redundancy.

The proposed PID does not suffer from this issue. Under the assumption of the Markov Condition, the bivariate tensors are used to infer the graph representing this system, after which the indirect associations can be removed [19], and the definitions for redundant and unique information applied. In this case it is immediately clear that there is no redundant contribution to *Z*: *X* and *Y* are statistically independent, therefore there is no path with a length greater than one. To illustrate how to use the data and the tensors to determine the probability distributions associated to a path, the steps that should be taken after the “pruning” is elaborated on. We start with determining the tensors constituting the path {X}{Y}{Z}, T{X}{Y} and T{Y}{Z}. From the distribution in Table 1 it follows that

$$T\left\{X\right\}\left\{Y\right\}=\left(\begin{array}{cc} \frac{1}{2} & \frac{1}{2}\\ \frac{1}{2} & \frac{1}{2} \end{array}\right),\;\mathrm{and}\;T\left\{Y\right\}\left\{Z\right\}=\left(\begin{array}{cccc} \frac{1}{2} & 0 & \frac{1}{2} & 0\\ 0 & \frac{1}{2} & 0 & \frac{1}{2} \end{array}\right).$$

Using Equation (5), we find that

$$T\left\{X\right\}\;\left\{Y\right\}\;\left\{Z\right\}=\left(\begin{array}{cccc} \frac{1}{4} & \frac{1}{4} & \frac{1}{4} & \frac{1}{4}\\ \frac{1}{4} & \frac{1}{4} & \frac{1}{4} & \frac{1}{4} \end{array}\right).$$

This tensor contains the transition probabilities, that is, the conditional probabilities $p_{1}\left(z|x\right)$ associated to the path {X}{Y}{Z}. Applying this tensor to the probability mass function for the input *X*, $p\left(x\right)\;=\;\left(\frac{1}{2},\frac{1}{2}\right)$, results in the pmf $p_{1}\left(z\right)\;=\;\left(\frac{1}{4},\frac{1}{4},\frac{1}{4},\frac{1}{4}\right)$ associated to the path {X}{Y}{Z}. All relevant probability distributions related to the path {X}{Y}{Z} are tabulated in Table 2.

Applying Equation (3) leads to $I_{p_{1}}\left(X;Z\right)\;=\;0$. Because $R\left(X,Y;Z\right)\;=\;\min\left[I_{p_{1}}\left(X;Z\right),I_{p_{2}}\left(Y;Z\right)\right]$, we find that in this case there is no redundant information information in *Z*. In general however, the structure needs to be inferred first because an in indirect association could be induced via a cascade. This is (also) illustrated in the next example.

### 3.2. Negative Contributions

Rauh et al. [22] demonstrated that the left monotonicity property and the identity property are incompatible with non-negative partial information components for systems comprising over three nodes, that is, the *local positivity* property is violated. In this section it is demonstrated that for the proposed PID, negative contributions are also possible for systems comprising three variables. We furthermore offer a potential explanation as to why negative contributions can arise in these cases.

#### 3.2.1. Negative Synergistic Contributions Due to Unobserved Common Causes?

**Figure 2:**
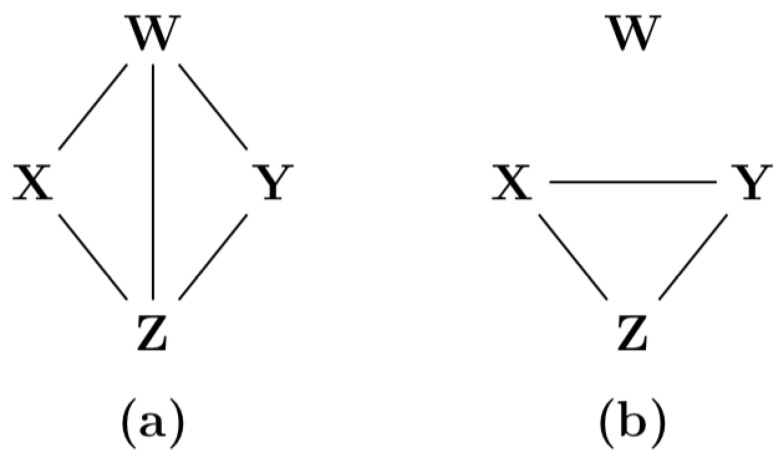
(**a**) The graph when all variables are observed. (**b**) The graph when *W* is not observed.

A result of the proposed definitions is that an unobserved common cause can lead to a negative synergistic contribution. In Table 3 an example is given of such a system. The related graphs are depicted in Figure 2. In Appendix H it is shown that in there is no path between *X* and *Y* in case *W* is observed.

If the common cause *W* is observed, the path {X}{Y} does not exist when the tensor of this path equals the tensor of the path {X}{W}{Y}.

Using Table 4 and Equation (3), we find that R(X,Y;Z)≤ 0.0271 bit in case the common cause is not observed. In Reference [6] it is shown that

(26) I(X;Y|Z)−I(X;Y)=S(X,Y;Z)−R(X,Y;Z).

The left-hand side is the interaction information [2]. Because this equals −0.1226 bit, the synergistic information is negative as per Equation (26).

From Equation (26), it follows that S(X,Y;Z)=I(X;Y|Z)−I(X;Y)+R(X,Y;Z), that is, S(X,Y;Z)<0 when

(27) I(X;Y|Z)−I(X;Y)<−R(X,Y;Z).

A necessary but insufficient condition is that left-hand side of this equation is negative. This can, for example, be the case when an unobserved common cause for the sources also transfers information to the target directly. To illustrate the potential diagnostics utility of a negative synergistic contribution, we investigate the system comprising three variables as tabulated in Table 5. The proposed path-based PID results in a negative synergistic term, implying that there must be a fourth, unobserved variable. Given the binary nature of the variables, we should be able to infer the *boolean machinery*. Table 5 also shows an equivalent but observational sufficient system comprising four variables. The variables in the three parameter system can be fully explained by boolean operations on the unobserved variable.

The reader can verify that in this case, because X,Y, and *Z* are functions of *W*, we can conclude that the association between *X* and *Z*, and the association between *Y* and *Z* are spurious.

In this section it was demonstrated that negative synergistic information can be induced in the case of an unobserved common cause. More research is needed to determine if this is the only cause for negative synergy in a system comprising three variables, and if so, how this could be used in practice.

#### 3.2.2. Negative Contributions Due to Overestimation of Unique Information

**Figure 3:**
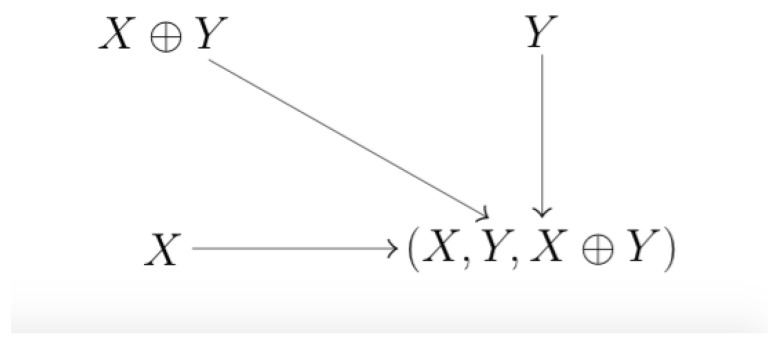
The graph for the system used to demonstrate that the Left Monotonicity property and the identity property are incompatible with the local positivity property.

The system used by Rauh et al. [22] comprises three source nodes: *X*, *Y*, and X⊕Y, with ⊕ the symbol for the Exclusive Or operator. The sources *X* and *Y* are independent identically distributed binary variables. The target node is defined as (X,Y,X⊕Y). In Figure 3 the underlying graph is depicted. This structure follows directly from the independence of *X* and *Y*, and from the probability distribution of the Xor. While in the previous example negative arose due to an *underdetermined system*, that is, a system with unobserved variables, we hypothesize that negativity in this case is due to an *over determined* system.

The system in Figure 3 only contains direct paths. Irrespective of the contributions of the lower three levels in Figure 1b, the unique information components indicated by the nodes {1}, {2} and {3} add up to 3 bit, while the target can be described by 2 bit. This implies that there must be a negative contribution. Partial Information Decomposition does not forbid us to add an extra source that is independent from all other sources, and to add the same variable to the target. This will, per definition, lead to a negative contribution.

The two examples discussed in this section demonstrated that the proposed PID can result in negative information contributions. For the system comprising three variables, a negative synergistic contribution arose due to an unobserved variable. In the example of the system comprising four variables, a negative term arose due to addition of a variable that is independent of the other sources, to both the set of causes as the target. Whether these examples describe all causes of negative contributions is an open question that warrants future research.

### 3.3. Dyadic and Triadic Systems

Next we apply the proposed method to the two data sets from Table 6. Although these sets have different underlying dependency structures, they apparently have the same statistical structure [5].

For the dyadic set, the tensors are given by:

$$T\left\{X\right\}\left\{Y\right\}=\left(\begin{array}{cccc} \frac{1}{2} & 0 & \frac{1}{2} & 0\\ \frac{1}{2} & 0 & \frac{1}{2} & 0\\ 0 & \frac{1}{2} & 0 & \frac{1}{2}\\ 0 & \frac{1}{2} & 0 & \frac{1}{2} \end{array}\right),\;T\left\{X\right\}\left\{Z\right\}=\left(\begin{array}{cccc} \frac{1}{2} & \frac{1}{2} & 0 & 0\\ 0 & 0 & \frac{1}{2} & \frac{1}{2}\\ \frac{1}{2} & \frac{1}{2} & 0 & 0\\ 0 & 0 & \frac{1}{2} & \frac{1}{2}. \end{array}\right),$$

T{Y}{Z}=T{X}{Y},T{Y}{X}=T{X}{Z},T{Z}{Y}=T{X}{Z}, and T{Z}{X}=T{X}{Y}. Because no relation is the result of a cascade, for example, T{Y}{Z}≠T{Y}{X}·T{X}{Z}, the structure is that of an undirected triangle. Lets assume we are interested in the partial information decomposition of the total information in *Z*. The reader can confirm that all the elements of the tensor T{X}{Y}{Z} equal $\frac{1}{4}$—there is no redundant information contribution to *Z*.

For the triadic set, the tensor T{X}{Y} equals

$$T\left\{X\right\}\left\{Y\right\}=\left(\begin{array}{cccc} \frac{1}{2} & 0 & \frac{1}{2} & 0\\ 0 & \frac{1}{2} & 0 & \frac{1}{2}\\ \frac{1}{2} & 0 & \frac{1}{2} & 0\\ 0 & \frac{1}{2} & 0 & \frac{1}{2} \end{array}\right).$$

All other tensors are equal to T{X}{Y}. We again assume that *Z* is the target. Because T{X}{Y}{Z}=T{X}{Y}·T{Y}{Z}, the association between *X* and *Z* can be fully explained by the cascade X→Y→Z. Likewise, the association between *Y* and *Z* can be fully explained by the cascade Y→X→Z, that is, the structure differs from the structure underlying the dyadic set. Applying the proposed redundancy to both situations, we find that there is no unique information but only redundant information contributions in *Z* from both *X* and *Y*.

This example shows that the difference in underlying structure is reflected in two ways. First, the graphs related to the dyadic set and the triadic set are different. Second, for the dyadic set there is no redundant information, while for the triadic set, *Z* does contain redundant information. This result suggest that indeed (some of) the internal dynamics is captured when using tensors and path-based measures instead of aggregated measures.

### 3.4. Comparison with Other Measures

To get an idea about the behavior of the proposed path-based redundancy measure, we compared it with four other measures—(i) Pointwise Partial Information Decomposition, using the average Partial Information atoms 〈i〉, (ii) the redundancy measure proposed by Williams and Beer $I_{min}$, (iii) $I_{broja}$, the redundancy measure proposed in References [23,24], and (iv) the redundancy based on Pointwise Common Change in Surprisal, $I_{CCS}$ [11]. The proposed path-based redundancy measure is represented as $I_{△}$.

#### 3.4.1. Comparison with Pointwise Partial Information Decomposition

As mentioned in the introduction, Finn and Lizier proposed a partial decomposition based on pointwise mutual information, PPID [12]. The authors also introduce a new example called “Pointwise Unique”, where in any pointwise configuration only one source holds non-zero information about the target. While other proposed a priori aggregated based measures do not identify unique information for the so-called PwUnq distribution, their approach, the approach of Ince, and the approach proposed in a recent paper [25] does.

As with the previous examples, the probability distribution is used to determine the underlying structure. Although the procedure that needs to be followed is now clear, we specifically determine the tensor for the path {X}{Y}{Z} for the PwUnq distribution using Table 7 and Equation (5). Because

$$T\left\{X\right\}\left\{Y\right\}=\left(\begin{array}{ccc} 0 & \frac{1}{2} & \frac{1}{2}\\ 1 & 0 & 0\\ 1 & 0 & 0 \end{array}\right),\;\mathrm{and}\;T\left\{Y\right\}\left\{Z\right\}=\left(\begin{array}{cc} \frac{1}{2} & \frac{1}{2}\\ 1 & 0\\ 0 & 1 \end{array}\right),\;T\left\{X\right\}\left\{Y\right\}\left\{Z\right\}=\left(\begin{array}{cc} \frac{1}{2} & \frac{1}{2}\\ \frac{1}{2} & \frac{1}{2}\\ \frac{1}{2} & \frac{1}{2} \end{array}\right).$$

A communication channel with identical rows in the probability transition tensor is incapable of transmitting any information—the redundant information equals 0 bit. The final PID results for PwUnq distribution are tabulated in Table 8.

From this table it is evident that our proposed PID does give the “correct” decomposition for the PwUnq distribution. This implies that some aspects of the pointwise approach are captured by the path-based PID.

#### 3.4.2. Comparison with $I_{min}$, $I_{broja}$, and $I_{CCS}$

To get an idea about how the proposed PID compares to other PIDs, we applied the proposed PID, and three other PIDs, to the probability distributions in Table 9. This table contains some distributions from Reference [11].

For each distribution in Table 9, the resulting decomposition is tabulated in a dedicated table. Table 10 contains the decomposition for distribution 5A, Table 11 contains the decomposition for distribution 5B, Table 12 contains the decomposition for the 5C distribution, Table 13 contains the decomposition for the ReducedOr distribution, Table 14 contains the decomposition for the Xor distribution, Table 15 contains the decomposition for the And distribution, and Table 16 contains the decomposition for the Sum distribution.

An interesting but also worrying difference between other proposed methods and our proposed method is that there is no path-based redundancy in the And, and in the similar Or distributions. However, the proposed PID for And/Or does not contradict the relation between interaction information and synergistic and redundant information, S(X,Y;Z)−R(X,Y;Z) [6], the synergistic information is larger than the redundant information.

From these limited set of examples, it follows that the proposed PID is comparable to the other PIDs.

## 4. Discussion

In this article, we have shown that a partial information decomposition comprising non-negative unique and redundant contributions follows naturally from the framework of tensors when ergodicity, stationarity, and the Markov Condition are assumed. Because we introduced no new information theoretical measures, it is our contention that a partial information decomposition is possible within the framework of “classical” Shannon information theory for data for which these assumptions are valid. A partial information decomposition in terms of “aggregated measures” is problematic when no exact expressions for indirect paths can be determined. It reduces to a rather straightforward exercise when using a foundational aspect of information theory, communication channels and their tensor representation, leading to an additional mutual information measure: path-based mutual information. In this paper, some of the characteristics of this new information measure were explored. Based on this new measure, a path-based redundancy was defined. The main idea behind path-based redundancy is that unique information must come from a source that is capable to (also) transmit its data directly to the target, and that redundant information is therefore transferred via paths with length larger than one, and if there are more paths towards the that contain the exact same sources, the overall, shared information, is the minimum of the path-based mutual information for these paths.

An issue of concern is that the proposed PID deviates from all other proposed PIDs for the And and Or distributions. Additionally, it was demonstrated that the resulting path-based PID could lead to negative information components, and we speculated that this might have diagnostics utility. Apart from these aspects, it is in general an open question if, and if so, how, any PID can be used to diagnose a complex electromechanical system comprising hundreds of components. The number of partial information terms increases rapidly as a function of the number of variables, for example, there are more than $5\times 10^{22}$ partial information terms for a system comprising nine variables [6].

In conclusion, some aspects of the proposed PID warrant future research, the fact that it is path-based, and that the tensors can be used to simulate the behavior of the network, opens new avenues for trying to apply the PID to systems with a large number of components. Future research will therefore focus on the use of tensor algebra, graph theory and/or simulation to try to apply the PID to large systems. Although not mentioned explicitly in this paper, the path-based approach is also applicable to transfer entropy [26], an inherent asymmetric information theoretical measure that can be used to infer directionality of an association. Application of the path-based PID to transfer entropy will be addressed in a different paper.

## Figures and Tables

**Table 1:** Distribution for the “two-bit copy problem”.

*X*	*Y*	*Z*	*p (x, y, z)*
0	0	(0, 0)	1/4
0	1	(0, 1)	1/4
1	0	(1, 0)	1/4
1	1	(1, 1)	1/4

**Table 2:** Marginal, conditional and joint distributions for the cascade X→Y→Z, that is, the path {X}{Y}{Z}, with $p_{1}\left(z|x\right)\;=\;T\left\{X\right\}\;\left\{Y\right\}\;\left\{Z\right\}$, and $p_{1}\left(z\right)=p\left(x\right)\cdot T\left\{X\right\}\;\left\{Y\right\}\;\left\{Z\right\}$. For the pmf p(x) the index is not used because this pmf is independent of the chain.

Source *X*	*Target Z*	*p (x)*	$p_{1}\left(z\right)$	$p_{1}\left(z|x\right)$	$p_{1}\left(z,x\right)$	$p_{1}\left(z|x\right)$/$p_{1}\left(z\right)$
0	(0, 0)	1/2	1/4	1/4	1/8	1
0	(0, 1)	1/2	1/4	1/4	1/8	1
0	(1, 0)	1/2	1/4	1/4	1/8	1
0	(1, 1)	1/2	1/4	1/4	1/8	1
1	(0, 0)	1/2	1/4	1/4	1/8	1
1	(0, 1)	1/2	1/4	1/4	1/8	1
1	(1, 0)	1/2	1/4	1/4	1/8	1
1	(1, 1)	1/2	1/4	1/4	1/8	1

**Table 3:** Example of a system with an unobserved common cause. **(a)** Data set comprising three parameters. **(b)** Unobserved common cause. $X=W_{1}$, $Y=W_{1}\;OR\;W_{2}$, and $Z=W_{1}\;A\mathrm{ND}\;W_{2}$.

	(a) Observed Data			(b) Unobserved Common Cause	
***X***	***Y***	***Z***		$W=(W_{1},W_{2})$	p(x,y,z,w)
0	0	0		(0, 0)	1/4
0	1	0		(0, 1)	1/4
1	1	0		(1, 0)	1/4
1	1	1		(1, 1)	1/4

**Table 4:** Marginal, conditional and joint distributions for path {X}{Y}{Z}, with $p_{1}\left(z|x\right)\;=\;T\left\{X\right\}\;\left\{Y\right\}\;\left\{Z\right\}$, and $p_{1}\left(z\right)\;=\;p\left(x\right)\cdot T\left\{X\right\}\;\left\{Y\right\}\;\left\{Z\right\}$.

Source *X*	*Target Z*	p(x)	$p_{1}\left(z\right)$	$p_{1}\left(z|x\right)$	$p_{1}\left(z,x\right)$	$p_{1}\left(z|x\right)/p_{1}\left(z\right)$
0	0	1/2	3/4	5/6	5/12	10/9
0	1	1/2	1/4	1/6	1/12	2/3
1	0	1/2	3/4	2/3	4/12	8/9
1	1	1/2	1/4	1/3	2/12	4/3

**Table 5:** Negative synergistic information in a system comprising three variables, implies that there must be a system comprising four variables resulting in the same probability distribution when a common cause is unobserved. The four variable system consists of the unobserved common cause, *W*, $X\;=\;W_{1}$ And $W_{2}$, Y= (Not $W_{2}$) And $W_{1}$, and $Z\;=\;W_{2}$.

3 variable system				4 Variable System			
*X*	*Y*	*Z*	*p (x, y, z)*	$W=(W_{1},W_{2})$	$W_{1}$ And $W_{2}$	$W_{1}$And (Not $W_{2}$)	$W_{2}$
0	0	0	1/4	(0, 0)	0	0	0
0	0	1	1/4	(0, 1)	0	0	1
0	1	0	1/4	(1, 0)	0	1	0
1	0	1	1/4	(1, 1)	1	0	1

**Table 6:** Two systems, both comprising three random variables with identical joint probabilities per combination of the random variables. The underlying structures are very different, which can be seen when the variables are represented in two bits, for example, the binary expansion for X=3 equals $X_{0}X_{1}\;=\;11$. **(a)** For the dyadic (pair-wise) set, $X_{0}\;=\;Y_{1}$,$Y_{0}\;=\;Z_{1}$, and $Z_{0}\;=\;X_{1}$. **(b)** For the triadic (three-way) set, $X_{0}+Y_{0}+Z_{0}$ mod2, and $X_{1}+Y_{1}+Z_{1}$.

	(a) Dyadic						(b) Triadic		
***X***	***Y***	***Z***	***p (x, y, z)***			***X***	***Y***	***Z***	***p (x, y, z)***
0	0	0	1/8			0	0	0	1/8
0	2	1	1/8			1	1	1	1/8
1	0	2	1/8			0	2	2	1/8
1	2	3	1/8			1	3	3	1/8
2	1	0	1/8			2	0	2	1/8
2	3	1	1/8			3	1	3	1/8
3	1	2	1/8			2	2	0	1/8
3	3	3	1/8			3	3	1	1/8

**Table 7:** The probability distribution for PwUnq.

*X*	*Y*	*Z*	*p (x, y, z)*
0	1	1	1/4
1	0	1	1/4
0	2	2	1/4
2	0	2	1/4

**Table 8:** Partial information decomposition (PID) for PwUnq. The partial information decomposition for redundant {1}{2}, unique, {1} and {2}, and synergistic information {12}. With $I_{\partial }\left(\langle i\rangle \right)$ the value for the specific atom, using the average partial information, and $I_{\partial }\left(I_{△}\right)$ the partial information atom based on path-based mutual information.

Lattice Node	$I_{\partial }\left(\langle i\rangle \right)$	$I_{\partial }\left(I_{△}\right)$
{12}	0	0
{2}	0.5	0.5
{1}	0.5	0.5
{1} {2}	0	0

**Table 9:** The probability distributions from Reference [12]. We left out the distributions for And and Or because these are considered to be well known.

		5A			5B			5C			ReducedOr			Xor			Sum		
*X*	*Y*		*Z*	*p (x, y, z)*		*Z*	*p (x, y, z)*		*Z*	*p (x, y, z)*		*B*	*p (x, y, z)*		*B*	*p (x, y, z)*		*Z*	*p (x, y, z)*
0	0		0	1/3		0	1/4		0	1/6		0	1/2		0	1/4		0	1/4
0	1		1	1/3		1	1/4		1	1/6		1	1/4		1	1/4		1	1/4
1	0		2	1/3		2	1/4		2	1/6		1	1/4		1	1/4		1	1/4
1	1		−	−		1	1/4		0	1/6		−	−		0	1/4		2	1/4
0	1		−	−		−	−		1	1/6		−	−		−	−		0	−
1	1		−	−		−	−		1	1/6		−	−		−	−		0	−

**Table 10:** PID for 5a.

Lattice Node	$I_{\partial }\left(I_{\min}\right)$	$I_{\partial }\left(I_{\mathrm{broja}}\right)$	$I_{\partial }\left(I_{\mathrm{CCS}}\right)$	$I_{\partial }\left(I_{△}\right)$
{12}	0.3333	0	0.1383	0.1383
{2}	0.3333	0.6666	0.5283	0.5283
{1}	0.3333	0.6666	0.5283	0.5283
{1} {2}	0.5850	0.2516	0.3900	0.3900

**Table 11:** PID for 5b.

Lattice Node	$I_{\partial }\left(I_{\min}\right)$	$I_{\partial }\left(I_{\mathrm{broja}}\right)$	$I_{\partial }\left(I_{\mathrm{CCS}}\right)$	$I_{\partial }\left(I_{△}\right)$
{12}	0.5	0	0	0
{2}	0.5	1	1	1
{1}	0	0.5	0.5	0.5
{1} {2}	0.5	0	0	0

**Table 12:** PID for 5c.

Lattice Node	$I_{\partial }\left(I_{\min}\right)$	$I_{\partial }\left(I_{\mathrm{broja}}\right)$	$I_{\partial }\left(I_{\mathrm{CCS}}\right)$	$I_{\partial }\left(I_{△}\right)$
{12}	0.67	0.67	0.67	0.67
{2}	0.25	0.25	0.25	0.25
{1}	0	0	0	0
{1} {2}	0	0	0	0

**Table 13:** PID for ReducedOr.

Lattice Node	$I_{\partial }\left(I_{\min}\right)$	$I_{\partial }\left(I_{\mathrm{broja}}\right)$	$I_{\partial }\left(I_{\mathrm{CCS}}\right)$	$I_{\partial }\left(I_{△}\right)$
{12}	0.69	0.69	0.38	0.40
{2}	0	0	0.31	0.29
{1}	0	0	0.31	0.29
{1} {2}	0.31	0.31	0	0.02

**Table 14:** PID for Xor.

Lattice Node	$I_{\partial }\left(I_{\min}\right)$	$I_{\partial }\left(I_{\mathrm{broja}}\right)$	$I_{\partial }\left(I_{\mathrm{CCS}}\right)$	$I_{\partial }\left(I_{△}\right)$
{12}	1	1	1	1
{2}	0	0	0	0
{1}	0	0	0	0
{1} {2}	0	0	0	0

**Table 15:** PID for And/Or.

Lattice Node	$I_{\partial }\left(I_{\min}\right)$	$I_{\partial }\left(I_{\mathrm{broja}}\right)$	$I_{\partial }\left(I_{\mathrm{CCS}}\right)$	$I_{\partial }\left(I_{△}\right)$
{12}	0.5	0.5	0.29	0.19
{2}	0	0	0.21	0.31
{1}	0	0	0.21	0.31
{1} {2}	0.31	0.31	0.10	0

**Table 16:** PID for Sum.

Lattice Node	$I_{\partial }\left(I_{\min}\right)$	$I_{\partial }\left(I_{\mathrm{broja}}\right)$	$I_{\partial }\left(I_{\mathrm{CCS}}\right)$	$I_{\partial }\left(I_{△}\right)$
{12}	1	1	0.5	0.5
{2}	0	0	0.5	0.5
{1}	0	0	0.5	0.5
{1} {2}	0.5	0.5	0	0

## Appendix A. Proof of General Validity of Equation (4)

**Proof.** Let *Z* be dependent on *X*, *Y*, and on a set of unobserved variables *U*. As per law of total probability, $p^{z}\;=\;\sum_{x,y,u}p^{xyu}p_{xyu}^{z}$. “Summing out” *x* and *u* results in $p^{z}\;=\;\sum_{y}p^{y}B_{y}^{z}$. In a similar fashion, it can be shown that for this system $p^{y}\;=\;\sum_{x}p^{x}A_{x}^{y}$, and $p^{z}\;=\;\sum_{x}p^{x}C_{x}^{z}$. Substituting the expression for $p^{y}$ in the expression for $p^{z}$ results in $p^{z}\;=\;\sum_{x}p^{x}\sum_{y}A_{x}^{y}B_{y}^{z}$. Combining this with $p^{z}\;=\;\sum_{x}p^{x}C_{x}^{z}$, results in Equation (4). □

## Appendix B. Proof of Lemma 1

**Proof.** Let

(A1) $$I\left\{X\right\}\left\{Y\right\}\left\{Z\right\}=\sum_{x,z}p^{xz}\log_{2}\left[\frac{\sum_{y}A_{x}^{y}B_{y}^{z}}{p^{z}}\right].$$

Because $A_{x}^{y}\;=\;A_{y}^{‡x}p^{y}/p^{x}$, and $B_{y}^{z}\;=\;B_{z}^{‡y}p^{z}/p^{y}$, Equation (A1) can be rewritten as

$$I\left\{X\right\}\left\{Y\right\}\left\{Z\right\}=\sum_{x,z}p^{xz}\log_{2}\left[\frac{\sum_{y}B_{z}^{‡y}A_{y}^{‡x}}{p^{x}}\right],$$

that is, I{X}{Y}{Z}=I{Z}{Y}{X}. □

## Appendix C. Proof of Equation (22)

**Proof.** First of all we notice that without loss of generality, Equation (9) leads to the following inequality

(A2) I{13}{t}≥I{1}{t}.

Adding a new source to both sides of this inequality, does not change the inequality,

(A3) I{12}{13}{t}≥I{12}{1}{t}.

Next we notice that, again without loss of generality, Equation (9) also leads to

(A4) I{12}{1}≥I{1}{1}.

Adding a new destination to both sides of the inequality, does not change this inequality either,

(A5) I{12}{1}{t}≥I{1}{1}{t}.

Combining Equations (A3) and (A5) and using I{1}{1}{t}=I{1}{t}, leads to the inequality

(A6) I{12}{13}{t}≥I{1}{t}.

□

## Appendix D. Proof of Generalized Self-Redundancy (Theorem 2)

**Proof.** Let T{X}{Z}=C, T{Y}{Z}=B, and T{X}{Y}=A. Because *X* and *Y* are equivalent, it follows immediately that

I{X}{Y}{Z}=I(X;Z),andI{Y}{X}{Z}=I(Y;Z).

To proof equality of I(X;Z) and I(Y;Z), we use Equation (3), and Equation (6) to express the difference I(X;Z)−I(Y;Z) as

(A7) $$I\left(X;Z\right)-I\left(Y;Z\right)=\sum_{x,z}p^{xz}\log_{2}\left[\sum_{y^{\prime }}A_{x}^{y^{\prime }}B_{y^{\prime }}^{z}\right]-\sum_{y,z}p^{yz}\log_{2}\left[B_{y}^{z}\right].$$

Because A is a stochastic permutation tensor, there is only one non-zero entry per row and per column. We can therefore define $\sum_{y^{\prime }}A_{x}^{y^{\prime }}B_{y^{\prime }}^{z}\;:=\;B_{y(x)}^{z}$, where the function y(x) returns the column of the permutation tensor containing the non-zero entry as a function of row *x* of the permutation tensor. This function is invertible. Using this new index, Equation (A7) can now be rewritten as

(A8) $$I\left(X;Z\right)-I\left(Y;Z\right)=\sum_{x,z}p^{y(x)}B_{y(x)}^{z}\log_{2}\left[B_{y(x)}^{z}\right]-\sum_{y,z}p^{y}B_{y}^{z}\log_{2}\left[B_{y}^{z}\right].$$

Both sums on the right-hand side are equal, so the difference evaluates to zero. □

## Appendix E. Proof of Path-Based Redundancy Inequality Theorem 3

Using Definition 2 and Lemma 1, it is proven that within a system comprising three variables there is a specific relation between the redundancies within this system.

**Proof.** Using Definition 2, we get

$$\begin{array}{cc} R(X,Y;Z) & =\min\left[I\{X\}\{Y\}\{Z\},I\{Y\}\{X\}\{Z\}\right],\\ R(Z,Y;X) & =\min\left[I\{Z\}\{Y\}\{X\},I\{Y\}\{Z\}\{X\}\right],\\ R(Z,X;Y) & =\min\left[I\{Z\}\{X\}\{Y\},I\{X\}\{Z\}\{Y\}\right]. \end{array}$$

Applying Lemma 1 and using α:=I{X}{Y}{Z}, β:=I{Y}{X}{Z}, γ:=I{Y}{Z}{X}, $R_{1}\;\;=\;\;R\left(X,Y;Z\right)$, $R_{2}\;=\;R\left(Z,Y;X\right)$, and $R_{3}\;=\;R\left(Z,X;Y\right)$, this set of redundancies can be simplified to:

(A9) $$\begin{array}{cc} R_{1} & :=\min\left[\alpha ,\beta \right], \end{array}$$

(A10) $$\begin{array}{cc} R_{2} & :=\min\left[\alpha ,\gamma \right], \end{array}$$

(A11) $$\begin{array}{cc} R_{3} & :=\min\left[\beta ,\gamma \right]. \end{array}$$

In Table A1 the (in)equalities for all possible combinations following from Equation (A9) are taulated.

**Table A1:** All possible combinations for the redundancies from Equation (A9).

$R_{1}$	$R_{2}$	$R_{3}$	Conclusion
α	α	β	$R_{3}\geq R_{1}=R_{2}$
α	α	γ	$R_{3}\geq R_{1}=R_{2}$
α	γ	β	$R_{1}\geq R_{2}=R_{3}$
α	γ	γ	$R_{1}\geq R_{2}=R_{3}$
β	α	β	$R_{2}\geq R_{1}=R_{3}$
β	α	γ	$R_{2}\geq R_{1}=R_{3}$
β	γ	β	$R_{2}\geq R_{1}=R_{3}$
β	γ	γ	$R_{1}\geq R_{2}=R_{3}$ From this table it follows that

(A12) $$R\left(X,Y;Z\right)\geq \min\left[R(Z,Y;X),R(Z,X;Y)\right].$$

□

## Appendix F. Proof of Identity Property Equation (23)

**Proof.** Per definition

$$R\left(X,Y;XY\right)=\min\left[I\{X\}\{Y\}\{XY\}\;,I\{Y\}\{X\}\{XY\}\right].$$

Assume that, in index notation, $p^{x}$ equals the pmf of *X*, $p^{y}$ equals the pmf of *Y*, and $p^{xy}$ equals the pmf of XY. Assume furthermore that $A_{x}^{y}$ are the tensor elements for the path {X}{Y}. Per definition $A_{y}^{‡x}$ represent the tensor elements for the path {Y}{X}. Let tensor elements for the path {X}{XY} equal $C_{x}^{xy}$, and the tensor elements for the path {Y}{XY} equal $B_{y}^{xy}$. Using these tensors, the path-based redundancy equals

$$R\left(X,Y;XY\right)=\min\left[\sum_{x,y}p^{xy}\log_{2}\left[\frac{\sum_{y^{\prime }}A_{x}^{y^{\prime }}B_{y^{\prime }}^{xy}}{p^{xy}}\right],\sum_{x,y}p^{xy}\log_{2}\left[\frac{\sum_{x^{\prime }}A_{y}^{‡x\prime }C_{x\prime }^{xy}}{p^{xy}}\right]\right].$$

The tensor elements $B_{y}^{xy}$ are related to $A_{y}^{‡x}$. It follows immediately that $B_{y^{\prime }=y}^{xy}=A_{y}^{‡x}$ and $B_{y^{\prime }\neq y}^{xy}=0$, Likewise, $C_{x^{\prime }=x}^{xy}=A_{x}^{y}$ and $C_{x^{\prime }\neq x}^{xy}=0$. We can rewrite the joint probabilities as $p^{xy}=p^{x}A_{x}^{y}$, and as $p^{xy}=p^{y}A_{y}^{‡x}$. Using these expressions, we rewrite the equation for the path-based redundancy as

$$R\left(X,Y;XY\right)=\min\left[\sum_{x,y}p^{xy}\log_{2}\left[\frac{A_{y}^{‡x}}{p^{x}}\right],\sum_{x,y}p^{xy}\log_{2}\left[\frac{A_{x}^{y}}{p^{y}}\right]\right].$$

Because both sums represent I(X;Y), the mutual information between *X* and *Y*, the proposed path-based redundancy measure satisfies the identity property. □

## Appendix G. Proof of Left Monotonicity (see Equation (24))

For the proof of the left monotonicity, the Redundancy lattice of Figure 1b, Theorem 3, the symmetry axiom, the monotonicity axiom, and the generalized self-redundancy axiom are used.
**Proof.** We distinguish two situations. *X* and *Y* are equivalent, and *X* and *Y* are not equivalent.

### Appendix G.1. Equivalence of X and Y

First we notice that in this case the generalized self-redundancy axiom applied to $R(X,Y;Z_{1})$ results in

(A13) $$R\left(X,Y;Z_{1}\right)=I\left(X;Z_{1}\right).$$

The generalized self-redundancy axiom applied to $R(X,Y;Z_{1}Z_{2})$ results in

(A14) $$R\left(X,Y;Z_{1}Z_{2}\right)=I\left(X;Z_{1}Z_{2}\right).$$

As per symmetry and monotonicity axioms,

(A15) $$R\left(X,Y;Z_{1}Z_{2}\right)\geq I\left(Z_{1};X\right).$$

Combining Equations (A13) and (A15) gives us

$$R\left(X,Y;Z_{1}Z_{2}\right)\geq R\left(X,Y;Z_{1}\right).$$

### Appendix G.2. Non-Equivalence of X and Y

In this section, we use the shorthand notation from Appendix E, that is, $R(X,Y;Z_{1})$ is written as $R_{1}$, $R(Z_{1},Y;X)$ as $R_{2}$, and $R(Z_{1},X;Y)$ as $R_{3}$. Where equivalence implied that $R_{2}\;=\;R_{3}$, non-equivalence implies that $R_{2}\;\neq \;R_{3}$ (Corollary 1)

Applying Theorem 3 to the path-based redundancy $R(X,Y;Z_{1}Z_{2})$ results in

(A16) $$R\left(X,Y;Z_{1}Z_{2}\right)\geq \min\left[R\left(Z_{1}Z_{2},Y;X\right),R\left(Z_{1}Z_{2},X;Y\right)\right].$$

Replacing $Z_{1}$ with 1, $Z_{2}$ with 2, *Y* with 3, or *X* with 3, it follows from Figure 1b that the inequality of Equation (A16) gives rise to the inequality

(A17) $$R\left(X,Y;Z_{1}Z_{2}\right)\geq \min\left[R\left(Z_{1},Y;X\right),R\left(Z_{1},X;Y\right)\right].$$

We now apply Theorem 3 to the right-hand side of Equation (A17).

(A18) $$R\left(X,Y;Z_{1}Z_{2}\right)\geq \min\left[R\left(X,Y;Z_{1}\right),R\left(X,Z_{1};Y\right),R\left(Y,X;Z_{1}\right),R\left(Y,Z_{1};X\right)\right].$$

Using the symmetry axiom and using the earlier mentioned shorthand notation we get

(A19) $$R\left(X,Y;Z_{1}Z_{2}\right)\geq \min\left[R_{1},R_{2},R_{3}\right].$$

From Table A1 it follows that in case $R_{2}\neq R_{3}$,

(A20) $$R\left(X,Y;Z_{1}Z_{2}\right)\geq R\left(X,Y;Z_{1}\right).$$

□

## Appendix H. Demonstration of the Non-Existence of a Path between *X* and *Y* in Case *W* is Observed Section 3.2.1

See Figure 2 for reference. Using Table 3, the reader can verify that T{X}{Y}=T{X}{W}·T{W}{Y}, because

$$T\left\{X\right\}\left\{Y\right\}=\left(\begin{array}{cc} \frac{1}{2} & \frac{1}{2}\\ 0 & 1 \end{array}\right),\;T\left\{X\right\}\left\{W\right\}=\left(\begin{array}{cccc} \frac{1}{2} & \frac{1}{2} & 0 & 0\\ 0 & 0 & \frac{1}{2} & \frac{1}{2} \end{array}\right),\;\mathrm{and}\;T\left\{W\right\}\left\{Y\right\}=\left(\begin{array}{cc} 1 & 0\\ 0 & 1\\ 0 & 1\\ 0 & 1 \end{array}\right).$$

This implies that the path {X}{Y} can be fully explained by the indirect path {X}{W}{Y}. However, if the common cause is unobserved (see Figure 2b), there is no reason to state that the path is nonexistent. Using Table 3 we find that

$$T\left\{X\right\}\left\{Z\right\}=\left(\begin{array}{cc} 1 & 0\\ \frac{1}{2} & \frac{1}{2} \end{array}\right),T\left\{Z\right\}\left\{Y\right\}=\left(\begin{array}{cc} \frac{1}{3} & \frac{2}{3}\\ 0 & 1 \end{array}\right),\;\mathrm{and}\;T\left\{X\right\}\left\{Z\right\}\cdot T\left\{Z\right\}\left\{Y\right\}=\left(\begin{array}{cc} \frac{1}{3} & \frac{2}{3}\\ \frac{1}{6} & \frac{5}{6} \end{array}\right),$$

that is, T{X}{Y}≠T{X}{Z}·T{Z}{Y}. In a similar fashion it follows that T{Y}{X}≠T{Y}{Z}·T{Z}{X}. This means that the structure is a fully connected system and that redundant information is contributed via the paths {X}{Y}{Z} and {Y}{X}{Z}.
